# What’s in a text-to-image prompt? The potential of stable diffusion in visual arts education

**DOI:** 10.1016/j.heliyon.2023.e16757

**Published:** 2023-05-26

**Authors:** Nassim Dehouche, Kullathida Dehouche

**Affiliations:** a Business Administration Division, Mahidol University International College, Salaya, Thailand; b Poh-Chang Academy of Arts, Rajamangala University of Technology Rattanakosin, Bangkok, Thailand

**Keywords:** Artificial intelligence, Art, Education, Computational creativity, Intellectual property

## Abstract

Text-to-Image artificial intelligence (AI) recently saw a major breakthrough with the release of Dall-E and its open-source counterpart, Stable Diffusion. These programs allow anyone to create original visual art pieces by simply providing descriptions in natural language (prompts). Using a sample of 72,980 Stable Diffusion prompts, we propose a formalization of this new medium of art creation and assess its potential for teaching the history of art, aesthetics, and technique. Our findings indicate that text-to-Image AI has the potential to revolutionize the way art is taught, offering new, cost-effective possibilities for experimentation and expression. However, it also raises important questions about the ownership of artistic works. As more and more art is created using these programs, it will be crucial to establish new legal and economic models to protect the rights of artists.

“It is, in the first place, 'by a word conceived in intellect' that the artist, whether human or divine, works.” Ananda K. Coomaraswamy [1].

## 1 Introduction

The traditional view of art, espoused by Coomaraswamy [1], is that of (human) art as imitation (of divine creation), with the word as a starting point. This view, notably challenged by contemporary expressionist and formalist perspectives [2], was given a new technical expression with recent advances in artificial intelligence (AI).

Indeed, AI has made impressive strides in the realm of creativity, with computers now able to generate relevant and original text [3] and images [4,5], in response to simple natural language prompts. Some of these outputs have even been indistinguishable from human creations, leading to their recognition in traditional art contests [6].

AI-generated art remains a controversial topic, with notable debates over whether it can truly be considered art in the first place [7], but despite the increasing academic interest in generative AI models, little attention has been given to their potential use in visual arts education. In our view, these models contain a compressed version of centuries of human artistic creations, which presents an undeniable interest for art education. Thus, in this paper, we explore the possibilities of incorporating them in visual art education, particularly for the teaching of art history, aesthetics, and technique.

Following this introductory section, the remainder of this paper is organized as follows. Section 2 situates recent developments in the field of Text-to-Image in the broader history of AI-generated art. Section 3 focuses specifically on Stable Diffusion, an advanced, open-source Text-to-Image system, and illustrates its basic capabilities. Section 4 describes the methods and data of our analysis of 72,980 Stable Diffusion interactions. Based on this analysis, Section 5 presents our results and proposes a formalization and procedural framework for Stable Diffusion prompts that can serve as a basis for their formal usage in educational software or curricula, and discusses some of its potential uses for the teaching of subjects such as the history of art, aesthetics, and technique, as well as its implications for the protection of the intellectual property of artists. Section 6 describes an example of a still life photography exercise using Stable Diffusion. Section 7 discusses some of the possible risks and limitations of integrating Text-to-Image software in visual arts education. Lastly, Section 6 concludes this paper by outlining the work that remains to be done, in our view, to facilitate the integration of Text-to-Image AI in art education.

## 2 A brief history of AI-generated art

The first attempts at using Artificial Intelligence to create coherent, original content from human prompts can be traced back to the 1950s, when researchers at the MIT Artificial Intelligence Laboratory created a program called ELIZA [8]. ELIZA was able to generate simple responses to text input, using pattern matching and natural language processing techniques. While not strictly art, ELIZA was an early example of Text-to-Text: software that could generate original text output that was intended to be interpreted by humans. One of the first examples of AI-generated art proper was a program called AARON, developed by artist Harold Cohen in the 1970s [9]. AARON was a computer program that was capable of generating complex drawings and paintings. AARON used a set of rules and constraints to create its art, and was able to learn from its own outputs to improve over time.

As AI technology advanced in the 1980s and 1990s, more complex and sophisticated AI-generated art began to emerge. For instance, Karl Sims generated unique 3D images and animations based on evolutionary algorithms [10]. In recent years, the advent of deep learning has led to even more realistic outputs, and consequently, AI-generated art gained increasing attention from both the art world and the general public. In 2015, a team at Google used deep learning techniques to train a neural network on a dataset of over 10,000 paintings, with the goal of generating original works of art from input images. The resulting program, known as DeepDream [11], was able to create surreal, visually striking images from input images (Image-to-Image). Another notable example is the work of a Paris-based art collective named "Obvious," which resulted in a software-generated portrait that sold for over $432,000 at a Christie's auction, in 2018 [12].

Year 2020 saw a major qualitative leap in Text-to-Text capabilities, with the release of the third generation Generative Pretrained Transformer (GPT-3), by private research firm OpenAI [3]. GPT-3 constitutes an important advance in terms of the generality of Text-to-Text models, and is able to generate text that is highly coherent, in response to virtually any prompt in natural language. This was made possible by the sheer size of the model, which consisted of 175 billion parameters; an order of magnitude more than the second largest similar model to date. This vast number of parameters allowed GPT-3 to comprehend language tasks it was not particularly trained for, and ushered in the era of Large Language Models. These models have the ability to generate high-quality, human-like text, which can be used in a variety of applications, including machine translation, text summarization, and creative writing. The success of GPT-3 led to the development of CLIP [13], another breakthrough model by OpenAI, which was designed to link text to images. CLIP (Contrastive Language–Image Pretraining) is a general-purpose image-text model trained on 400 million text-image pairs from the internet, allowing it to perform image classification with any user-provided label. It can also generate text that accurately describes any input image (Image-to-Text). Based on these advances, OpenAI released DALL-E [4], which is able to generate convincing images from text descriptions (Text-to-Image). While DALL-E remains a proprietary, closed-source software, the code of CLIP was released open-source. This allowed artificial intelligence firm Stability AI to develop and train Stable Diffusion [5], an open-source Text-to-Image model, with comparable performance to DALL-E. Stable Diffusion was released under a permissive license allowing commercial and non-commercial usage.

Although they represent an important technical breakthrough, CLIP, and the Text-to-Image systems based on it, also raise important ethical and societal concerns. Because of its training on mass, indiscriminate internet data, CLIP has a propensity to reproduce biased and unfair stereotypes present in culture and society [13], and its possible unfair usage of protected works has alerted legal experts [14]. These systems also have the potential to be used for nefarious purposes, such as creating fake news or spreading misinformation [15].

## 3 Stable diffusion

Stable Diffusion is a text-to-image model, released in 2022, that uses a deep learning technique called latent diffusion [5] to generate images based on text descriptions. Unlike some previous Text-to-Image models, Stable Diffusion's code and model weights are publicly available and can be run on most consumer hardware.

To generate images, Stable Diffusion uses CLIP [12] to project a text prompt into a joint text-image embedding space, and select a rough, noisy image that is semantically close to the input prompt. This image is then subject to a denoising method based on the latent diffusion model to produce the final image. In addition to a text prompt, the Text-to-Image generation script within Stable Diffusion allows users to input various parameters such as sampling type, output image dimensions, and seed value.

**Fig. 1:**
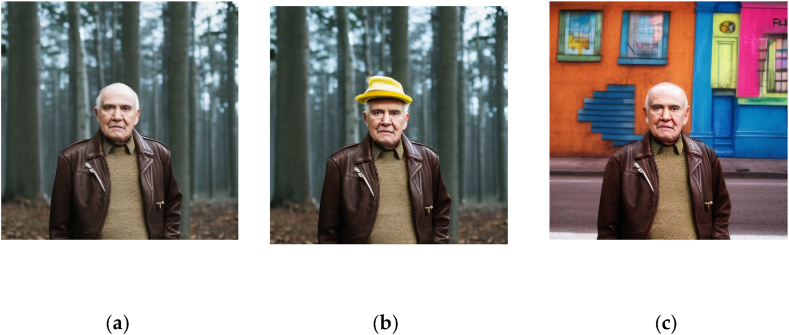
Images generated in Stable Diffusion 2.1., with the prompts “detailed photograph of an older man wearing a leather jacket, waist shot, forest background, in the style of Brandon Stanton, Humans of New York”. Additional inpainting was applied to generate Figures (b) and (c).

**Fig. 2:**
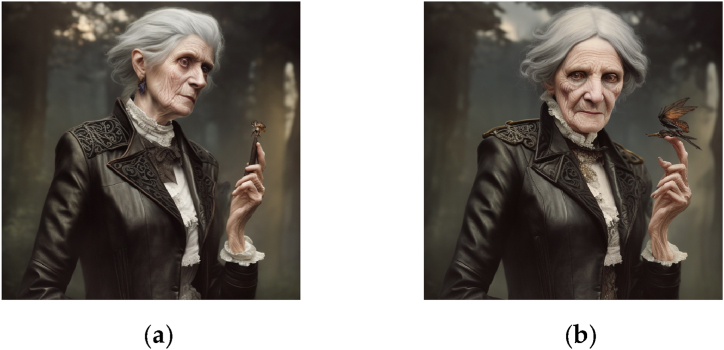
Images generated in Stable Diffusion 2.1., with the prompts “digital illustration of an older woman wearing a leather jacket, Victorian aesthetics, waist shot, forest background, in the style of Magali Villeneuve”.

This integer parameter is typically set randomly, but a constant seed value allows for reproducibility and the conservation of certain image aspects across prompts. For example, using a constant seed in Fig. 1(a) and (b) conserves some facial features across prompts. A constant seed can also maintain a subject's appearance in different poses and settings, as shown in Fig. 2(a) and (b).

However, even with a constant seed value, text prompts can generate random artifacts and imperfections, which may necessitate post-processing. Some front-end implementations of Stable Diffusion, like DreamStudio, offer post-processing functions such as inpainting and outpainting. Inpainting alters a specific image part by filling in a masked area based on a user-provided prompt, while outpainting extends an image beyond its original dimensions. Both functions use the Stable Diffusion model to generate new content. For instance, starting with Fig. 1(a), we can add accessories or change the background with respective inpainting prompts, as shown in Fig. 1(b) and (c).

Moreover, Fig. 1, Fig. 2 Illustrate Stable Diffusion’s ability to reproduce the style of contemporary, practicing artists (photographer Brandon Stanton and illustrator Magali Villeneuve, respectively). This controversial aspect of generative AI [16] is analyzed more thoroughly in Section 5.3.

## 4 Data and methods

Stable Diffusion’s output images are highly sensitive to the wording of text prompts, so we set out to examine the format and semantic content of this form of input. To this end, we gathered a dataset of 72,980 Stable Diffusion prompts from Lexica,^1^ a search engine that features curated Stable Diffusion outputs submitted by users along with the prompts that generated them. We conducted our analysis in three steps:

1. Tokenization: Each prompt is broken down into “tokens”; atomic linguistic terms, which can be words, phrases, symbols, or other meaningful elements of the prompt. This step is performed using the BERT Tokenizer [17] (Appendix 1).
2. Topic extraction: The goal of this step is to automatically identify the main topics or themes present in the 72,980 prompts, with the prior knowledge that they represent detailed descriptions of images. This is performed using the GPT-3 [3] API^2^ (Appendix 3).
3. Classification: Tokens, from each prompt, are classified into one or several of the linguistic topics identified in step 1, using the GPT-3 API (Appendix 4).

Additionally, the ability of Stable Diffusion to accurately reproduce the style of specific artists, whose work was used for its training, has been a controversial issue. To specifically examine the usage of this feature in prompts, in Section 7.2, we identified tokens that represent the name of an artist, brand, or collective using BERT's named-entity recognition function [17] and calculated the frequency of each of these entities in the 72,980 prompts under consideration (Appendix 2).

## 5 Results and discussion

### 5.1 Formalizing stable diffusion prompts

Topic extraction allows us to identify the primary elements (i.e. semantic categories of tokens) described in Table 1. These are the most frequent categories of keywords in the 72,980 considered prompts.

**Table 1:** Primary elements in 72,980 Stable Diffusion prompts.

Topic	Description
Subject	The characters and objects in the image, such as “a cyborg”, “two dogs”, “a car”, “a wizard”, etc.
Medium	The type of visual object that is the image, such as “digital illustration”, “photograph”, “3D render”, “concept art”, “poster”, etc.
Technique	The tools and software used to create the image, such as “Blender”, “pincushion lens”, “Unreal engine”, “Octane”, etc.
Genre	Aesthetic features that describe the artistic genre of the image, such as “anime”, “surreal”, “baroque”, “photorealistic”, sci-fi, black and white, epic fantasy, film noir, etc.
Mood	Features that describe the atmosphere and emotions of the image, such as “beautiful”, “eerie”, “bleak”, etc.
Tone	Features that describe the chromatic composition of the image, such as “pastel”, “synthwave colors”, “ethereal colors”, etc.
Lighting	The use of light and shadows in the image “dark”, "cinematic lighting", "realistic shaded lighting", "studio lighting", radiant light, etc.
Resolution	Features that describe the level of detail of the image, e.g. "highly-detailed", "photorealistic", "100 mm'', “8K”, “16K”, “HQ”, “sharp focus”, etc.
Artistic References	Artists or works of art to use as inspiration, e.g. “Greg Rutkowski”, “Studio Ghibli”, “Artgerm”, “Zaha Hadid”, etc.
Reception/Popularity	Awards, recognition, or trending status on art-focused platforms,. e.g. "trending on artstation", “masterpiece”, "award-winning”, etc.

Less frequent topics, that are extensions or additional details of the previous main topics are listed in Table 2.

**Table 2:** Secondary elements in 72,980 Stable Diffusion prompts.

Topic	Examples
Physical attributes of the subject	race, age, clothing, accessories, “cute”, “glamorous”, “chonky”, etc.
Emotional or psychological traits of the subject	“happy”, “anxious”, “triumphant”, “pensive”, etc.
Environment/Setting	time, weather, “medieval”, “post-apocalyptic”, etc.
Symmetry/Repetition	“symmetry”, “symmetrical”, “pattern”, “motif”, “fractal”, etc.
Depth of field	“blurred background”, “deep focus”, “aperture”, “F/4”, “F/2.8, "sharp focus", "bokeh", etc.
Angle	“ultra wide angle”, “zenith view”, “cinematic view”, “close up”, etc.
Message/Meaning	“propaganda”, “religious’, “advertisement”, etc.

### 5.2 Proposed procedural classification

**Fig. 3:**
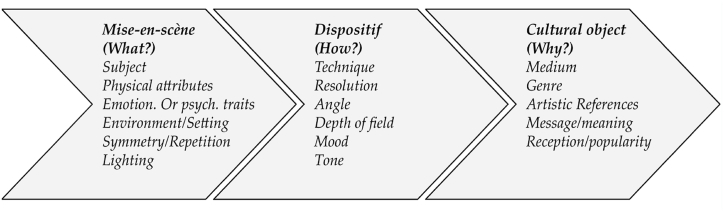
Proposed creative process for Text-to-Image prompts based on the semantic elements in 72,980 Stable Diffusion prompts.

The identified prompt elements align remarkably well with traditional photography concepts, and can be procedurally classified as in Fig. 3. This alignment suggests that the identified elements encompass key aspects of photography, including composition, lighting, subject matter, and style. By incorporating these elements into the prompts, we can enhance the ability of the model to generate images that adhere to established photography principles. Additionally, this categorization provides a clear structure for understanding and interpreting the relationships between various elements, which can further inform the design of prompts and improve the overall quality of the generated images.

**Fig. 4:**
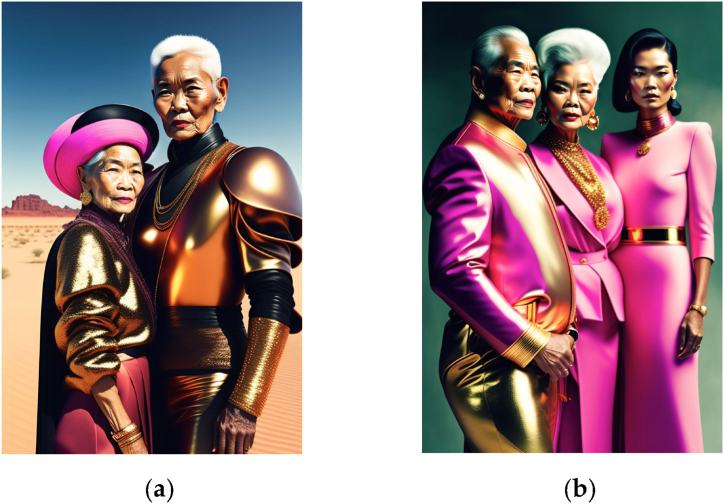
Images generated in Stable Diffusion 2.1., with the prompts “Fashion photograph of older Thai models wearing futuristic Thai clothes, pink and gold tones, radiant light, by Andrey Yakovlev, in (a) a desert setting (b) a studio setting”

**Fig. 5:**
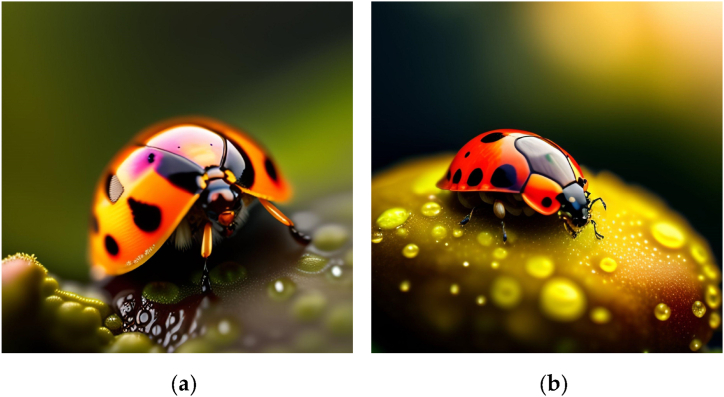
“Ladybug on a rainy leaf, forest background, close up, Macro lens, wide aperture, 8K, sharp edges”.

**Fig. 6:**
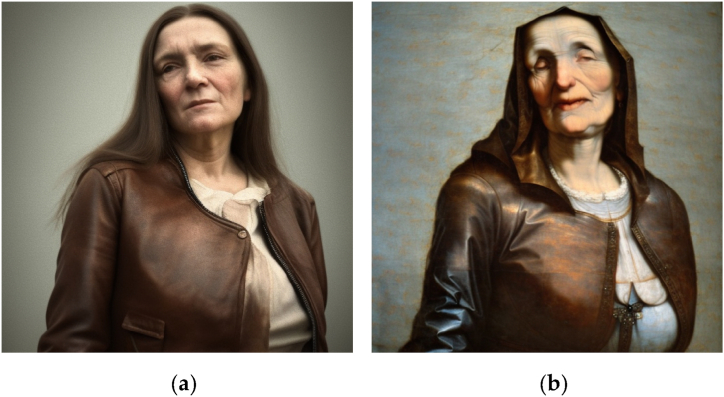
“Portrait of an older woman wearing a leather jacket, religious, award-winning”, as (a) “a photograph by Annie Leibovitz” and (b) “a renaissance painting by Michelangelo”.

● **M*ise-en-scène****:* Mise-en-scène is a term commonly used in the study of photography, film, and theater to refer to the arrangement of objects, settings, and actors within a shot or scene [18]. This category thus includes the visual and compositional elements that will appear in the frame to create the intended cultural object, e.g. “Fashion photograph of elegant older Thai models wearing futuristic Thai clothes, pink and gold tones, radiant light, by Andrey Yakovlev, in a desert setting/studio setting” illustrated in Fig. 4(a) and (b).
● ***Dispositif:*** In photography and film, the concept of dispositif pertains to the configuration of the material technology [19] used to capture an image. Within our more general classification, this category can also possibly include software tools and post-processing techniques for digital images. If mise-en-scène is *what* is displayed in the image, the dispositif would be *how* it is created, e.g. “close up, Macro lens, wide aperture, 8K, sharp edges”, illustrated in Fig. 5(a) and (b).
● ***Cultural object:*** These elements describe the “object” of the artist’s creation, understood in its double meaning of “artifact” and “purpose”; the latter understanding includes descriptions of the medium and genre of the image, as well as its positioning in the history of art through artistic references (e.g. “a photograph by Annie Leibovitz” or “a renaissance painting by Michelangelo”); the former descriptions of the message/meaning and reception/popularity (e.g. “religious, award-winning”). These two combinations of prompts are illustrated in Fig. 6(a) and (b).

It is important to note that the elements in our proposed procedural classification are not independent or exclusive. For example, using an artist's name as an artistic reference can influence the mood and tone of the resulting image. It can be interesting to explore unusual or conflicting combinations of these elements for creative purposes, but it is worth remembering that the initial image associated with a text by CLIP is a noisy pixel soup, and the prompts are meant to guide its denoising. Therefore, the more coherent the prompt, the better the outcome. Mastering Text-to-Image involves understanding the interplay of these elements, which includes a degree of randomness, in order to generate the most coherent art.

### 5.3 Quantitative results

Unsupervised topic modeling is a subjective task, making it challenging to objectively evaluate the model's performance due to the absence of ground truth labels [20]. Despite these limitations, diversity and coherence can be utilized to evaluate the composition of extracted topics. Diversity measures the variety of topics among classified tokens, while coherence evaluates the semantic relatedness of tokens to the topics, indicating how well the model captures the data's underlying structure. These indicators are evaluated using the script in Appendix 4.

Formally, diversity is the entropy of the distribution of tokens within a topic [21]. A higher diversity score indicates a more diverse range of words within a topic, while a lower score indicates that the topic is dominated by a few words. The diversity score is calculated using the formula:

Diversity = -Σ(P(x) * log2(P(x)))

Where P(x) represents the probability of each unique token x in the topic.

The diversity score ranges from 0 to log2(N), where N is the number of unique tokens in the topic. The minimum value of 0 is achieved when there's only one unique token (no diversity), and the maximum value of log2(N) is reached when all tokens are equally distributed. We then normalize this score as percentage, by dividing by log2(N).

The coherence score used in this application is calculated using the "c_v" measure provided by the Gensim library.^3^ Coherence is a measure of how well the words within a topic are semantically related. A higher coherence score indicates that the words within a topic are more likely to co-occur and have similar meanings, making the topic more interpretable. The "c_v" coherence measure is based on the sliding window approach with a cosine similarity measure. It calculates the pairwise cosine similarity between the word embeddings of the top words in a topic and averages these values to produce the coherence score. The coherence score ranges from −1 to 1. A score close to 1 indicates high coherence, meaning that the words within a topic are more likely to co-occur and share similar meanings. A score close to −1 indicates low coherence, meaning that the words within a topic are less likely to co-occur and share similar meanings. A score of 0 implies that there is no relationship between the words within a topic.

We report evaluation results for the tokenization, topic extraction and classification in Table 3, with a breakdown of different token subsets within the dataset (i.e. the topics/themes of our procedural classification) and their corresponding diversity and coherence scores.

**Table 3:** Quantitative indicators for topic modeling.

Token subset	Number of tokens	Diversity	Coherence	% of tokens
Full dataset	964,391	N/A	N/A	100%
Subject	173976	0.7872	0.135	17.04%
Medium	100779	0.2613	0.531	8.45%
Technique	70690	0.423	0.481	6.33%
Genre	64421	0.357	0.289	6.68%
Mood	26328	0.4681	0.174	2.73%
Tone	14948	0.370	0.567	1.55%
Lighting	102515	0.404	0.510	5.63%
Resolution	25074	0.3769	0.623	2.60%
Artistic References	183427	0.2279	0.371	19.02%
Reception/Popularity	77248	0.1837	0.651	8.01%
Physical attributes of the subject	11862	0.593	0.116	1.23%
Emotional or psychological traits of the subject	6944	0.423	0.348	3.72%
Environment/Setting	42530	0.623	0.244	10.41%
Symmetry/Repetition	4629	0.4067	0.470	0.48%
Depth of field	15430	0.364	0.583	1.6%
Angle	27292	0.190	0.563	2.83%
Message/Meaning	14948	0.364	0.201	1.55%

Unsurprisingly, the "Subject" subset of tokens presents the highest diversity score (0.7872) but a relatively low coherence score (0.135), suggesting that this subset contains a wide range of words that may not be semantically related, whereas the "Environment/Setting" subset has a relatively high diversity score (0.623) and a moderate coherence score (0.244), suggesting that it contains a variety of words, some of which may be semantically related.

The "Reception/Popularity" and "Resolution" subsets have relatively high coherence scores (0.651 and 0.623, respectively), indicating that the words within these subsets are more likely to co-occur and share similar meanings. While the low diversity of 0.1837 in the “Reception/Popularity” can be explained by its nature, we also observe a relatively low diversity score of 0.190 for the “Angle” subset of tokens, indicating that this prompt element is relatively under-explored.

**Fig. 7:**
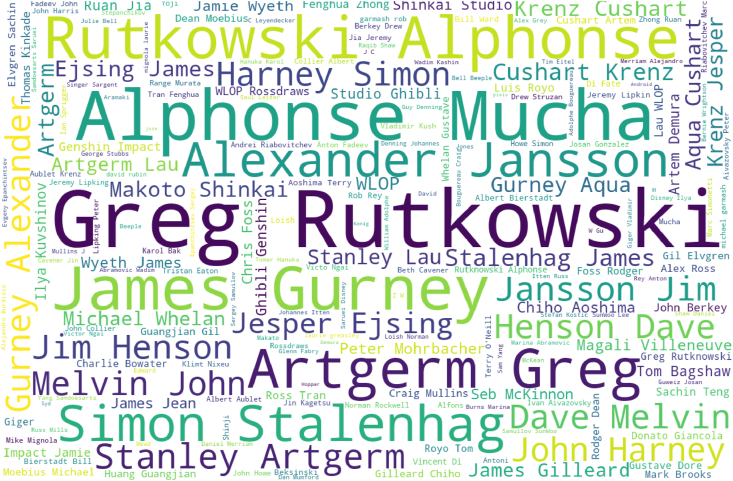
Word cloud of artists, brands, or collective names used for inspiration in 72,980 Stable Diffusion prompts.

Moreover, We observe that the "Artistic References" subset of tokens contains the highest percentage of tokens (19.02%) and the second-lowest diversity score (0.2279), indicating that the words in this subset are concentrated around a few key terms. The word cloud in Fig. 7., constructed using the Named Entity Recognition script in Appendix 2 shows the frequency of named entities used as artistic references in the 72,980 prompts under consideration. We found that these named entities predominantly refer to contemporary, practicing artists who frequently post their work on digital art platform ArtStation. For example, Polish painter Greg Rutkowski, including slight misspellings of his name and mentions alongside other artists, appears in 41.06% of the prompts, while mentions of ArtStation as an element of Reception/Popularity appear in 63.35% of the prompts.

## 6 Example classroom application: storytelling through Duality in Still Life Photography

In this exercise for a still life photography class, students are tasked with creating a pair of photographs for a hypothetical magazine cover. The objective is to portray a controversial topic (e.g. genetically-modified organisms, job automation, cryptocurrency, abortion, etc.) in both a favorable and unfavorable light, and Importantly, with an identical seed and only minimal variations in the mise-en-scène elements of the prompts generating the still life photographs.

**Fig. 8:**
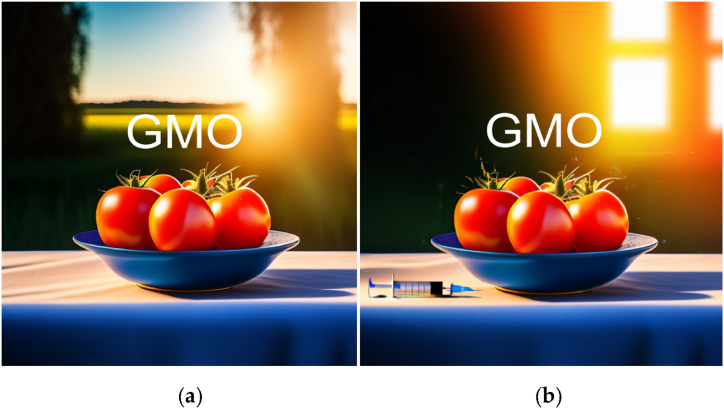
“A bowl of tomatoes sitting on top of a table, radiant light, warm colors, product photography by Jonathan Knowles, pixabay award-winning, stock photo, high dynamic range”, (a) “beautiful farm background” and (b) “next to a syringe, inside a secret research facility”. The characters “GMO” were added in post processing

For example, on the topic of Genetically-Modified Organisms (GMO), the two photographs in Fig. 8 Feature an identical bowl of tomatoes. The photograph in Fig. 8(a) is set against the radiant light of an outdoors farm, conveying a sense of abundance; and a blue sky, a sense of prosperity. In contrast, the photograph in Fig. 8(b) is staged indoors, to evoke secrecy, with a syringe on the table, suggesting the concerns and fears associated with GMOs.

**Objectives:** The "Duality in Still Life Photography" exercise challenges students to delve into the complexities of controversial topics and learn how to use composition as a visual storytelling technique, to present different perspectives. The constraint of minimal variations in the prompts aims to develop the artist's intentionality, as they must carefully consider how to manipulate the constituting elements of an image to convey different messages and emotions around the same topic. By exploring both the positive and negative aspects of these topics, students will gain a deeper understanding of the issues at hand and learn to communicate nuanced ideas through the composition of their still life photography.

Procedure:

● Students will choose a controversial topic from a list provided by the instructor or propose their own topic for approval.
● Research the chosen topic to gain an understanding of the various viewpoints and arguments surrounding the issue.
● Plan and compose two still life photographs using Stable Diffusion, that represent the topic in a favorable and unfavorable light, with minimal variations to their mise-en-scène elements.
● Edit and prepare the final images for presentation.
● Write a brief artist's statement explaining the choices made in each photograph, the intended message, and the techniques used to convey that message.

**Assessment:** Students are evaluated on their ability to:

● Demonstrate a clear understanding of the controversial topic and its complexities.
● Create visually compelling photographs that effectively communicate the intended messages.
● Utilize Mise-en-scène elements, such as Subject, Environment/Setting, symmetry/Repetition, Lighting to influence the viewer's perception.
● Articulate their creative process and choices in their artist's statement.

## 7 Risks and limitations

Despite its promising potential, a range of pedagogical and societal risks must be thoughtfully examined prior to incorporating Stable Diffusion into educational environments.

### 7.1 The risk of delegating artistic intentionality

A technical bottleneck in the development of artistic agency in machines remains the open problem of automating aesthetics evaluation, which still requires humans-in-the-loop [22,23]. An incomplete, practical heuristic to enhance the quality of generated images is to incorporate "Reception/Popularity" element in prompts, such as "award-winning" or "trending on Artstation." However, Stable Diffusion remains unable to assess the aesthetic quality of its output. Because of this limitation and its dependency on Large Language Models trained at the level of word-image associations, Stable Diffusion’s “understanding” of art remains superficial and essentially situated at the level of gimmicks (which, as noted by Ref. [24], remain "capitalism's most successful aesthetic category"). Consequently, a potential risk of the integration of text-to-image AI in visual arts classrooms lies, in our view, in delegating artistic intentionality to the system [25], thus constraining the range of expressions available to visual arts students, which can result in a homogenization of artistic expression, as Stable Diffusion draws from a fixed set of pre-defined intentions and styles rooted in centuries of human art history. Consequently, the potential for truly original and innovative works of art may be stifled, as artists become more inclined to adhere to the AI-generated "gimmicks" rather than pushing the boundaries of their own creativity. Consequently, it is important for educators to emphasize the theoretical distinction between “categorical intention”, that is the intention to create a piece of art with certain attributes and the more elusive “meaning-intention”, i.e. the artist's intention to elicit a particular interpretation or response from their audience [26]. As a prosaic example, using the term "propaganda poster" in a prompt, generates aesthetics tied to specific historical periods and geographies where such objects were stereotypically used. However, if an artist's meaning-intention is contemporary political propaganda, they would avoid using such literal terminology and instead explore more subtle and dynamic ways to convey their intention. To this end, and using the framework presented in Section 5.1., students should develop a heightened sensitivity to the terminology used in the "Message/Meaning" category. By carefully considering the combination of terms used and their relationship to the other elements of their artistic prompts, students should strike a balance between embracing the assistance offered by Stable Diffusion and maintaining their unique artistic vision.

### 7.2 The ethicality of stable diffusion

Legal clarity regarding the use of copyrighted material in the training of Stable Diffusion is a crucial prerequisite for its ethical implementation in educational settings. As the system possesses the capability to reproduce the work of practicing living artists, it raises concerns over potential copyright infringements and the unauthorized use of such materials. The popularity of some keywords found as artistic references in Fig. 7 Can be attributed to the fact that platforms such as *ArtStation*^4^ encourage artists to include detailed labels describing their work in order to make it more accessible to persons with disabilities, which makes these creations particularly useful for training Text-to-Image AI models. Thus, ArtStation artists are somehow penalized for their virtue. The legal question of whether this training constitutes plagiarism is still open [14] and may take years, if not decades, to be resolved. In addition to possible unfair usage of the intellectual property of these artists, the widespread use of Stable Diffusion also leads to the original creations of these artists being overshadowed in search engine results by AI-generated works that bear their names in the prompts.

While incomplete, as it does not account for works that are used implicitly in the creation of an image, a simple technical solution to these issues could be to devise compensation models for artists based on the frequency of their names appearing as a Style Reference in commercial Text-to-Image applications, similar to music streaming economic models.

### 7.3 The automation risk

Stable Diffusion and similar tools undoubtedly fuel concerns about the future of visual arts as a profession, among broader macroeconomic concerns about automation and the replacement of roles across industries by AI-powered systems. Somehow surprisingly, a recent, large-scale study conducted by OpenAI and others, on occupations with the highest exposure to AI-driven replacement found that Graphic Designers (along with e.g. Financial Managers, and Insurance Appraisers) were among the professions where AI could improve the productivity of workers, without necessarily replacing them, in contrast with professions such as Mathematicians or Blockchain Engineers, with a much higher exposure to automation. In this context, the role of "prompt engineer" has emerged as a possible profession of the future in various domains, including art and design. This new profession may offer new opportunities for visual arts graduates. As AI-generated artwork becomes more sophisticated, the importance of "book" knowledge of art (Art History, Aesthetics, Philosophy of Art) can see a resurgence, as it is becoming increasingly valuable in constructing prompts to guide the AI in generating images that meet specific artistic, historical, or cultural criteria, as well as evaluating the quality and creativity of its output.

## 8 Conclusion

This study aimed at developing a structured understanding of text-to-image prompts and connecting it to established art concepts, using topic extraction techniques.

Due to the unsupervised nature of this task, empirical validation is essential to confirm the effectiveness of the approach. We hope that visual arts educators will find value in our findings and methodology.

By bridging the gap between text-to-image prompts and traditional art concepts, we aim to facilitate the integration of these prompts into the educational and creative process. This connection could help educators and students to better understand the underlying structure of prompts, enabling them to design more effective and meaningful prompts for their own artistic endeavors. Furthermore, this structured understanding can contribute to the development of more advanced image generation models, which can better interpret and respond to prompts that are grounded in established art principles.

Stable Diffusion, while offering valuable assistance in the realm of visual arts, presents potential risks and limitations in terms of artistic intentionality and expression. However, with proper guidance and curation from educators, it can represent a valuable, didactic tool for the transmission of technical concepts, as well as more experiential concepts of artistic genres, movements, and aesthetics that characterize a cultural object. Additionally, variations on the elements of mise-en-scène and dispositif, for a constant seed integer, can constitute a fast and cheap method of experimentation and prototyping, before using costly studio time.

Notwithstanding their potential, for Stable Diffusion and similar software to be harmoniously integrated into the art world, it is necessary for there to be ethical and legal clarity surrounding the important questions they raise about the fair compensation for artists whose creations were used to train these models.

## Author contribution statement

Nassim Dehouche: Analyzed and interpreted the data; Wrote the paper.

Kullathida Dehouche: Conceived and designed the experiments; Performed the experiments; Analyzed and interpreted the data; Contributed reagents, materials, analysis tools or data; Wrote the paper.

## Additional information

A sample of 500 Stable Diffusion prompts used for this manuscript can be accessed on the Mendeley Data repository, at the following URL https://data.mendeley.com/datasets/sx9sfpm5hj/1 https://doi.org/10.17632/sx9sfpm5hj.1.

## Declaration of competing interest

The authors declare that they have no known competing financial interests or personal relationships that could have appeared to influence the work reported in this paper.

## Appendix 1 Tokenization and Topic Modeling

import pandas as pd

from transformers import BertTokenizer

import openai

# Loading the dataset

data = pd.read_csv("prompts.csv")

# Initializing the BERT tokenizer

tokenizer = BertTokenizer.from_pretrained("bert-base-uncased")

# Tokenizing each description

data["tokens"] = data["description"].apply(lambda x: tokenizer.tokenize(x))

# Replace with your API key

openai.api_key = "your-api-key"

# OpenAI API Setup

def generate_topics(n_topics):

prompt = f"Generate {n_topics} unique main topics for a dataset of descriptions of images."

response = openai.Completion.create(

engine="text-davinci-003",

prompt=prompt, max_tokens=200, n=1, stop=None, temperature=0.7,

)

topics = response.choices[0].text.strip().split(", ")

return topics

# Generating 20 main topics

n_topics = 20

main_topics = generate_topics(n_topics)

## Appendix 2 Named Entity Recognition

import pandas as pd

from transformers import pipeline

# Loading the CSV file

file_path = 'prompts.csv'

df = pd.read_csv(file_path)

# Initializing the Name Entity Recognition pipeline using BERT

ner_pipeline = pipeline("ner", model="dbmdz/bert-large-cased-finetuned-conll03-english")

# Function to extract entities representing artists, brands, or collectives

def extract_entities(prompt):

entities = ner_pipeline(prompt)

relevant_entities = []

for entity in entities:

if entity['entity'] == 'B-PER' or entity['entity'] == 'I-PER' or entity['entity'] == 'B-ORG' or entity['entity'] == 'I-ORG':

relevant_entities.append(entity['word'])

return relevant_entities

# Extracting entities from the dataset

entity_frequency = {}

for prompt in df['Prompt']:

entities = extract_entities(prompt)

for entity in entities:

if entity in entity_frequency:

entity_frequency[entity] += 1

else:

entity_frequency[entity] = 1

# Printing the frequency of each entity

for entity, count in entity_frequency.items():

print(f"{entity}: {count}")

## Appendix 3 Token Classification

def classify_tokens(tokens, main_topics):

prompt = f"Classify the following tokens into one of these main topics: {', '.join(main_topics)}.\n\nTokens: {', '.join(tokens)}\n\nTopic: "

response = openai.Completion.create(

engine="text-davinci-003",

prompt=prompt, max_tokens=20, n=1, stop=None, temperature=0.5,)

topic = response.choices[0].text.strip()

return topic

# Classifying tokens in topics

data["topic"] = data["tokens"].apply(lambda x: classify_tokens(x, main_topics))

## Appendix 4 Coherence and Diversity Evaluation

import numpy as np

from gensim.models.coherencemodel import CoherenceModel

from gensim.corpora.dictionary import Dictionary

# Computing the normalized diversity score

def diversity_score(topic):

unique_tokens, counts = np.unique(topic, return_counts=True)

total_tokens = len(topic)

probs = counts/total_tokens

max_diversity = np.log2(len(unique_tokens))

diversity = -np.sum(probs * np.log2(probs))/max_diversity

return diversity.

# Creating a list of tokenized descriptions for gensim

texts = data["tokens"].tolist()

# Creating a dictionary and corpus for gensim

gensim_dictionary = Dictionary(texts)

gensim_corpus = [gensim_dictionary.doc2bow(text) for text in texts]

# Computing the coherence and diversity scores for each topic

topic_scores = {}

for topic_name in main_topics:

topic_tokens = data[data["topic"] == topic_name]["tokens"].explode()

# Compute the normalized diversity score

diversity = diversity_score(topic_tokens)

# Get the top words for the topic

top_words = [gensim_dictionary[id] for id, _ in gensim_dictionary.doc2bow(topic_tokens)]

# Compute the coherence score

coherence_model = CoherenceModel(topics=[top_words], texts=texts, dictionary=gensim_dictionary, coherence="c_v")

coherence = coherence_model.get_coherence()

# Storing the scores

topic_scores[topic_name] = {"coherence": coherence, "diversity": diversity}

# Priting results

for topic_name, scores in topic_scores.items():

print(f"Topic: {topic_name}")

print(f"Coherence score: {scores['coherence']}")

print(f"Diversity score: {scores['diversity']}\n")

## Footnotes

1 https://lexica.art/.

3 https://pypi.org/project/gensim.

4 https://www.artstation.com.

## Footnotes

2 https://openai.com/api/.

## References

[1] Coomaraswamy A.K. (1956).

[2] Braembussche A. (2009).

[3] Brown T. (2020). Proceedings of the 33rd Conference on Neural Information Processing Systems (NeurIPS 2020).

[4] Ramesh A., Dhariwal P., Nichol A., Chu C., Chen M. (2021).

[5] Rombach R., Blattmann A., Lorenz D., Esser P., Ommer B. (2022). Proceedings of the IEEE Conference on Computer Vision and Pattern Recognition (CVPR).

[6] Roose L. (2022). https://www.nytimes.com/2022/09/02/technology/ai-artificial-intelligence-artists.html.

[7] Zylinska J. (2020).

[8] Weizenbaum J. (1976).

[9] Cohen P. (2016). Harold cohen and AARON. AI Mag..

[10] Sims K. (1992). Choreographed image flow. J. Vis. Comput. Animat..

[11] Mordvintsev A., Olah C., Tyka M. (2015). Deepdream-a code example for visualizing neural networks. Google Res..

[12] Cohn G. (2018). https://www.nytimes.com/2018/10/25/arts/design/ai-art-sold-christies.html.

[13] Dehouche N. (2021). Implicit stereotypes in pre-trained classifiers. IEEE Access.

[14] Franceschelli G., Musolesi M. (2022). Copyright in generative deep learning. Data & Policy.

[15] Fallis D. (2021). The epistemic threat of deepfakes. Philosophy Tech..

[16] Zeilinger M.A.I. (2021).

[17] Devlin J., Chang M.W., Lee K., Toutanova K. (2018).

[18] Sikov E. (2020). Film Studies.

[19] Kessler F., Chateau D., Moure J. (2016). Screens.

[20] Churchill, R., Singh, L. The Evolution of Topic Modeling. ACM Comput. Surv., 54(10), 1-35. https://dl.acm.org/doi/10.1145/3507900.

[21] Bérubé, N., Sainte-Marie, M., Mongeon, P., Larivière, V. Words by the tail: Assessing lexical diversity in scholarly titles using frequency-rank distribution tail fits. PLoS One 13(7): e0197775. 10.1371/journal.pone.0197775. PMC6037356 29985920

[22] Vera Nieto, D., Celona, L., Fernandez Labrador, C. Understanding Aesthetics with Language: A Photo Critique Dataset for Aesthetic Assessment. NeurIPS 2022: 36th Conference on Neural Information Processing Systems, New Orleans, USA.

[23] McCormack, J. Lomas, A. Understanding Aesthetic Evaluation Using Deep Learning. Artificial Intelligence in Music, Sound, Art and Design: 9th International Conference, EvoMUSART 2020, Held as Part of EvoStar 2020, Seville, Spain. 10.1007/978-3-030-43859-3_9.

[24] Ngai S. (2020).

[25] Gell A. (1998).

[26] Frank-Witt P. (2020). Intentionality in art: empirical exposure. J. Vis. Art Pract..

